# Capsule Endoscopy for Obscure Gastrointestinal Bleeding in Patients with Comorbid Rheumatic Diseases

**DOI:** 10.1155/2014/534345

**Published:** 2014-07-06

**Authors:** Neal Shahidi, George Ou, Jessica Tong, Ricky Kwok, Cherry Galorport, Joanna K. Law, Robert Enns

**Affiliations:** ^1^Department of Medicine, Division of Gastroenterology, University of British Columbia, Vancouver, BC, Canada V5Z 1M9; ^2^Department of Medicine, Division of Gastroenterology, Johns Hopkins University, Baltimore, MD 21205, USA; ^3^St. Paul's Hospital, University of British Columbia, Pacific Gastroenterology Associates, 770-1190 Hornby Street, Vancouver, BC, Canada V6Z 2K5

## Abstract

*Background and Aim*. We evaluated the association between patients with rheumatic diseases (RD) suffering from obscure gastrointestinal bleeding (OGIB) and positive capsule endoscopy (CE) findings. *Methods*. All CE procedures performed on patients with RD and OGIB were assessed from a large database at St. Paul's Hospital (Vancouver, BC, Canada) between December 2001 and April 2011. A positive finding on CE was defined as any pathology, including ulcers/erosions, vascular lesions, and mass lesions, perceived to be the source of bleeding. *Results*. Of the 1133 CEs performed, 41 (4%) complete CEs were for OGIB in patients with RD. Of these, 54% presented with overt bleeding. Mean age was 66 years. Positive findings were seen in 61% of patients. Ulcerations/erosions (36%) and vascular lesions (36%) were the most common findings. Significant differences between the RD versus non-RD populations included: inpatient status, nonsteroidal anti-inflammatory drug (NSAIDs) use, oral steroid use, and mean Charlson index score (all *P* ≤ 0.008). Similar nonsignificant trends were seen between positive and negative CEs among the RD population. *Conclusions*. The correlation between RD and positive CE findings is likely influenced by ongoing anti-inflammatory drug use, poorer health status, and a predisposition for angiodysplastic lesions.

## 1. Introduction

Capsule endoscopy (CE) is a novel diagnostic technique which allows assessment of the entire small bowel that is not feasible with conventional endoscopy. Its noninvasive nature together with its documented high sensitivity and specificity [1–3] has encouraged its use most notably in obscure gastrointestinal bleeding (OGIB). With OGIB comprising approximately 5% of all gastrointestinal bleeds, it represents a significant economic burden with multiple studies attempting to optimize investigational algorithms through cost-effectiveness analyses [4–7]. While identifying the source of OGIB early is characteristically uncommon, CE has led to an increased feasibility for identifying pathology allowing for superior patient outcomes, alongside a potential reduction in resource utilization [1, 2, 8] from repeated, expensive investigations.

With the emergence of CE as a pivotal tool in current investigational algorithms for OGIB, consequent studies [1, 8–11] have attempted to identify predictors of positive findings on CE with the goal of refining patient selection to optimize diagnostic yield. In a recent study assessing this correlation, we identified comorbid rheumatic diseases (RD) as a significant correlate to positive findings on CE [11]. While studies exist describing the association between gastrointestinal bleeding and RD, there is an overall deficit in the literature focusing on patients with RD suffering specifically from OGIB [12–14]. Therefore, we sought to further evaluate this correlation between patients with RD being evaluated for OGIB and positive pathology on CE. 

## 2. Methods

### 2.1. Population Description

All CE cases performed between December 2001 and April 2011 at St. Paul's Hospital (Vancouver, British Columbia, Canada) for the evaluation of OGIB in patients with comorbid RD were considered for inclusion. These cases were a specific subpopulation of a previous retrospective evaluation of all CE procedures performed for the evaluation of OGIB [11]. Written approval was obtained from our institutional ethics committee. Obscure gastrointestinal bleeding was defined as gastrointestinal bleeding with no apparent source subsequent to evaluation of the upper and lower gastrointestinal tracts by conventional endoscopic methods. Bleeding was stratified into overt bleeding (hematemesis, hematochezia, or melena) and occult bleeding (positive fecal occult blood test, iron deficiency anemia, or an acute drop in hemoglobin). Cases were excluded if patients had significant findings on conventional endoscopy and CE was undertaken to confirm that there were no other potential sources within the small bowel. The Charlson index [15, 16] was used as a framework for the definition of RD.

### 2.2. Capsule Endoscopy Procedure and Interpretation

Included CE procedures were performed using PillCam (Given Imaging, Yoqneam, Isreal), EndoCapsule (Olympus, Tokyo, Japan), or MiroCam (IntroMedic, Seoul, Korea). Informed consent was obtained prior to all CE procedures. All participants were instructed to stop oral iron supplementation 5 days prior to CE and to undergo bowel preparation which included adherence to a clear fluid diet and ingestion of 2 L of polyethylene glycol-electrolyte solution. Patients were allowed to drink and eat at 2 and 4 hours after ingestion of the capsule camera, respectively.

Subsequent to administration, the capsule assistant, the gastrointestinal therapeutics fellow, and a single experienced gastroenterologist reviewed each CE procedure independently with any discrepancy in findings being resolved by consensus. Positive findings on CE were defined as any definitive pathology (e.g., ulcers/erosions, vascular lesions, and mass lesions) perceived to be the source of bleeding. Fresh blood was also considered a positive finding as it suggests proximity of the lesion. In cases where multiple findings were identified, the most prominent lesion was recorded alongside its location. CE which (1) did not enter the small bowel, (2) traversed the small bowel for less than 1 hour in an unremarkable study, or (3) had excessive debris obscuring the examination warranting repeat procedure was considered incomplete.

### 2.3. Data Collection and Statistical Analysis

Data was extracted by two independent authors (NS, GO) retrospectively, with discrepancy being resolved by consensus with a third author (RE). Comorbid status was assessed utilizing definitions outlined by the Charlson Index [15, 16]. Identical definitions for covariables were utilized as in our previous study [11]. Further covariables were extracted including the use of selective serotonin reuptake inhibitors (SSRIs), immunomodulators (azathioprine, hydroxychloroquine, cyclosporine, penicillamine, gold therapy, leflunomide, methotrexate, minocycline, and sulfasalazine), and biologic therapies. Follow-up data were obtained in a retrospective manner up to 1 year after CE or after confirmatory testing.

Differences in demographic characteristics and comorbid disease states between both CE positive (CE+) and CE negative (CE−) cases among the RD population and between the RD and non-RD populations [11] were evaluated using Fisher's exact test, chi-square test, or *t*-test as appropriate. A *P* value of <0.05 was deemed to be statistically significant.

## 3. Results

A total of 1133 CE procedures were performed between December 2001 and April 2011, with 42 CE cases for the indication of OGIB in patients with comorbid RD. One case was incomplete which was subsequently excluded from analysis leaving 41 complete CE cases. The most common diagnoses were rheumatoid arthritis (*n* = 18), polymyalgia rheumatica (*n* = 6), scleroderma (*n* = 5), systemic lupus erythematosus (*n* = 5), and dermatomyositis (*n* = 2). Mean age was 66 years with 27% being male (Table 1). Most cases were completed on an outpatient basis (80%). Twenty-two patients presented with a history of overt bleeding, with melena being the most common overt symptomatic presentation. Among those who presented with occult bleeding, 11 tested positive for fecal occult blood, 7 were confirmed to simply have iron deficiency anemia, and 1 had an acute decline in hemoglobin with suspected gastrointestinal etiology. The majority of cases (78%) had symptoms for greater than 24 weeks. Concerning precapsule endoscopic assessment, the mean number of esophagogastroduodenoscopies (EGDs), enteroscopies, and colonoscopies was 1.8, 0.5, and 1.6, respectively. The most common comorbidities in this cohort included chronic pulmonary disease (15%), diabetes with or without end-organ complications (12% each), and moderate to severe renal disease (15%) (Table 2). As for medications, proton pump inhibitors (66%), immunomodulators (39%), oral steroids (29%), antiplatelet/coagulants (22%), nonsteroidal anti-inflammatory drugs (NSAIDs) (22%), and SSRIs (12%) were common. Biologic therapy was seen in 1 case. Only 27% of cases had no history of transfusion requirements.

Of the 41 complete CE procedures, 61% identified a definitive source of gastrointestinal bleeding (Table 3). The most common findings were erosions/ulcerations (36%) and angiodysplastic/vascular lesions (36%). Sources of pathology were frequently located in the small bowel (84%) as expected given negative precapsule evaluations. However, gastric pathologies were identified in 3 cases and cecal pathology in 1 case. No capsule related adverse events were seen but 6 CEs did not reach the cecum by the end of the camera battery life and 1 was retained in the stomach due to a stricture/lesion.

Following identification of positive findings, further intervention was recommended in 15/25 of cases. Follow-up data were available for 13/15 of cases with pathology confirmed in 8/13 of cases. This included 2 mass lesions and 1 ulcerated lesion at an anastomotic site which were surgically excised. Alternatively, 4 angiodysplastic lesions and 1 case of gastric antral vascular ectasia were identified on subsequent upper endoscopy and were treated with argon plasma coagulation. Follow-up data were available in all 8 cases subsequent to intervention for a mean of 10 months, with rebleeding occurring in 63% of cases.

Among the 10 CE+ cases where conservative management was recommended, follow-up was available in 3/10 of cases, over a mean of 6 months with 67% suffering from rebleeding. Concerning the CE− cases, all 16 cases underwent conservative management. Follow-up data were available among 10/16 of cases over a mean of 5 months with 60% suffering from rebleeding.

In comparing the RD versus non-RD populations, statistically significant differences were noted concerning male gender, inpatient status, mean Charlson index, NSAID use, oral steroid use, and proton pump inhibitor use (all *P* ≤ 0.008). Similar trends, albeit not statistically significant, were seen comparing the CE+ versus CE− RD cases concerning inpatient status, mean Charlson index, NSAID use, and oral steroid use.

## 4. Discussion

The evaluation of OGIB continues to become increasingly complex, with a myriad of costly diagnostic procedures. Consequently, not only does inappropriate diagnostic selection impair early diagnosis but also incurs increased costs placing onus on the refinement of current investigational algorithms. Recently, studies have surfaced evaluating the association of clinical and demographic factors with positive outcomes in CE [1, 8–11, 17]. With the recent emergence of RD as a novel significant predictor of positive findings, we sought to further elucidate this association. Our study shows that the correlation between RD and positive CE findings is influenced in a multifactorial manner; specifically, ongoing anti-inflammatory drug use, overall poorer health status, and a predisposition to vascular lesions may be pivotal factors driving this association.

Anti-inflammatory medications, specifically NSAIDs, are a cornerstone in the management of RD [18], with their gastrointestinal adverse effects well recognized in the literature [19, 20]. Specific to the small bowel, previously documented NSAID-induced lesions include erosions, ulcerations, mucosal diaphragms, strictures, and NSAID-associated enteropathy [21, 22]. As highlighted in our study, a potentially significant contributing factor for the correlation between RD and positive CE findings is the ongoing utilization of NSAIDs. Patients with comorbid RD were significantly more likely to consume NSAIDs compared to patients without RD suffering from OGIB described in our previous study (22% versus 4%, resp.) [11]. Furthermore, analyses within the RD subpopulation showed that regular NSAID use was more prevalent among CE+ cases (28%) compared to CE− cases (13%). Interestingly, NSAID use has not been shown in the literature to be a significant correlate with positive CE findings and this is surprising since it is well known that NSAIDs commonly result in small bowel mucosal breaks for which current methods of cytoprotection are not beneficial. However, perhaps the lack of reporting that NSAIDs increase the yield of positive findings on CE may be due to a lack of patient-reported NSAID use [23, 24].

In a recent study by Sidhu et al. [25] evaluating the surreptitious use of NSAIDs including aspirin in patients undergoing CE, 10/76 (14%) of patients were found to have urinalysis suggestive of NSAID use. Only 10% of these cases declared their utilization. Of the 10 cases with suspected NSAID use, 8 had positive findings on CE including erosions (*n* = 5), ulcerations (*n* = 2), and ulceration with early stricturing (*n* = 1). The findings by Sidhu et al. are supported by 3 CE+ cases within our study where suspected NSAID-induced pathology was identified despite patients' denial of regular NSAID use. This highlights the importance of rigorous questioning regarding the use of NSAIDs on history, specifically over-the-counter forms of these medications.

An interesting finding of our study is that multiple comorbid disease states (diabetes with end-organ damage, mild liver disease, and moderate to severe renal disease) were more prevalent not only within the RD population, but also on subanalyses among CE+ cases compared to CE− cases. A statistically significant difference was noted concerning the Charlson index as well as inpatient status. In isolation, inpatient status [10] and liver disease [17] have been shown to significantly correlate with positive CE findings on multivariate analyses. Comparable trends have been found for diabetes with end-organ damage [11], renal disease, [17] and Charlson index scores [11], albeit solely on univariate analyses. By all things in consideration, we feel that these isolated comorbid states may have played a potentially additive or synergistic role in conjunction with overall poorer health status within the RD population predisposing them to more readily apparent gastrointestinal lesions on CE.

Another potential factor driving the correlation between RD and positive CE findings was a predisposition to vascular/angiodysplastic lesions. Vascular lesions are common findings on CE, with a recent systematic review identifying angiodysplasia as the most prevalent finding on CE in patients with OGIB [3]. In our study, vascular lesions were the most common finding at 36%, which was equal to erosions/ulcerations. Notably, this was greater than the overall general population of OGIB cases (26%) extracted over the same time period [11]. Among those suffering from scleroderma, all positive findings were vascular/angiodysplastic in nature. This correlation between scleroderma and gastrointestinal vascular lesions has been documented in the literature [12], with case reports focusing on this relationship in patients with OGIB [13, 14].

Unfortunately, our study was not devoid of limitations. Specifically, it is retrospective in design and data were obtained from a single tertiary health centre. Furthermore, due to small sample size, statistical analysis that aimed at identifying significant correlations was limited, specifically when comparing CE+ versus CE− cases among the RD population. Lastly, our assessment of rebleeding after CE was limited, as our database was not initially designed to collect this data; therefore, it may overestimate the rebleeding risk given that patients without rebleeding were likely not commonly seen in follow-up.

In summary, optimization of current investigational algorithms through the identification of clinical correlates with positive CE findings is critical. In our study, we show that the previously identified correlation between RD and positive CE findings is likely driven by ongoing anti-inflammatory drug use, poor overall health status, and a predisposition to vascular lesions within the small bowel. This highlights the importance of minimizing the use of anti-inflammatory medications in this patient population as endoscopic and surgical management have limited benefit. Further analyses, specifically prospective studies that aim at further elucidating this correlation, are needed. Moreover, cost-effectiveness analyses are needed to assess the already identified correlates with positive CE findings to see whether further modification to diagnostic algorithms is warranted.

## Figures and Tables

**Table 1:** Demographic and clinical characteristics of included cases.

Characteristics	Negative CE (*N* = 16)		Positive CE (*N* = 25)		*P* value	RHEUM CE (*N* = 41)		Non-RHEUM CE (*N* = 627)		*P* value
	*N* (%)	SD	*N* (%)	SD		*N* (%)	SD	*N* (%)	SD	
Mean age (years)	64.50	17.05	67.68	9.59	0.449	66.44	12.91	63.19	15.26	0.183
Gender (male)	3 (18.8)		8 (32.0)		0.478	11 (26.8)		321 (51.2)		0.003
Status (inpatient)	2 (12.5)		6 (24.0)		0.448	8 (19.5)		42 (6.7)		0.008
Indication for CE					0.121					0.483
Occult	5 (31.3)		14 (56.0)			19 (46.3)		326 (52.0)		
Overt	11 (68.8)		11 (44.0)			22 (53.7)		301 (48.0)		
Occult breakdown										
Anemia	1 (20.0)		0 (0)			1 (5.3)		18 (2.9)		
IDA	3 (60.0)		4 (28.6)			7 (36.8)		130 (20.7)		
FOBT+	1 (20.0)		10 (71.4)			11 (57.9)		178 (28.4)		
Overt breakdown										
Hematemesis	1 (6.3)		0 (0)			1 (2.4)		9 (1.4)		
Hematochezia	4 (25.0)		4 (16.0)			8 (19.5)		124 (19.8)		
Melena	6 (37.5)		8 (32.0)			14 (34.2)		193 (30.8)		
Duration of problem					0.865					0.309
0–8 weeks	2 (12.5)		3 (12.0)			5 (12.2)		44 (7.0)		
9–24 weeks	2 (12.5)		2 (8.0)			4 (9.8)		51 (8.1)		
>24 weeks	12 (75.0)		20 (80.0)			32 (78.1)		532 (84.9)		
Mean EGDs	1.63	0.96	1.96	1.81	0.507	1.83	1.53	1.84	1.09	0.956
Mean enteroscopies	0.38	0.50	0.52	0.51	0.393	0.46	0.50	0.56	1.07	0.553
Mean colonoscopies	1.56	0.81	1.56	0.71	1.000	1.56	0.74	1.90	1.17	0.067
Excessive alcohol	0 (0)		1 (4.0)		1.000	1 (2.4)		43 (6.9)		0.510
Smoking	0 (0)		1 (4.0)		1.000	1 (2.4)		65 (10.4)		0.169
NSAIDs	2 (12.5)		7 (28.0)		0.441	9 (22.0)		23 (3.7)		<0.001
Antiplatelet/coagulation	4 (25.0)		5 (20.0)		0.717	9 (22.0)		97 (15.5)		0.271
Oral steroids	4 (25.0)		8 (32.0)		0.734	12 (29.3)		16 (2.6)		<0.001
Proton pump inhibitors	12 (75.0)		15 (60.0)		0.323	27 (65.9)		252 (40.2)		0.001
Number of transfusions					0.623					0.842
0 units	6 (37.5)		5 (20.0)			11 (26.8)		177 (28.2)		
1-2 units	1 (6.3)		3 (12.0)			4 (9.8)		68 (10.9)		
3–9 units	4 (25.0)		6 (24.0)			10 (24.4)		181 (28.9)		
≥10 units	5 (31.3)		11 (44.0)			16 (39.0)		201 (32.0)		

CE: capsule endoscopy; EGDs: esophagogastroduodenoscopies; FOBT+: fecal occult blood test positive; IDA: iron deficiency anemia; NSAIDs: nonsteroidal anti-inflammatory drugs; SD: standard deviation.

**Table 2:** Comorbid diseases of included cases.

Characteristics	Negative CE (*N* = 16)		Positive CE (*N* = 25)		*P* value	RHEUM CE (*N* = 41)		Non-RHEUM CE (*N* = 627)		*P* value
	*N* (%)	SD	*N* (%)	SD		*N* (%)	SD	*N* (%)	SD	
Myocardial infarction history	1 (6.3)		1 (4.0)		1.000	2 (4.9)		45 (7.2)		0.760
Congestive heart failure	0 (0)		1 (4.0)		1.000	1 (2.4)		59 (9.4)		0.164
Peripheral vascular disease	2 (12.5)		1 (4.0)		0.550	3 (7.3)		26 (4.2)		0.413
Cerebrovascular disease	1 (6.3)		1 (4.0)		1.000	2 (4.9)		47 (7.5)		0.760
Dementia	0 (0)		0 (0)		1.000	0 (0)		3 (0.5)		1.000
Chronic pulmonary disease	3 (18.8)		3 (12.0)		0.662	6 (14.6)		66 (10.5)		0.432
History of ulcer disease	2 (12.5)		2 (8.0)		0.637	4 (9.8)		53 (8.5)		0.771
Mild liver disease	0 (0)		4 (16.0)		0.143	4 (9.8)		21 (3.4)		0.060
Diabetes w/o end-organ damage	2 (12.5)		3 (12.0)		1.000	5 (12.2)		100 (16.0)		0.522
Hemiplegia or paraplegia	0 (0)		0 (0)		1.000	0 (0)		3 (0.5)		1.000
Diabetes w/end-organ damage	1 (6.3)		4 (16.0)		0.632	5 (12.2)		48 (7.7)		0.362
Moderate-severe renal disease	1 (6.3)		5 (20.0)		0.376	6 (14.6)		60 (9.6)		0.280
Any tumor	3 (18.8)		4 (16.0)		1.000	7 (17.1)		96 (15.3)		0.762
Leukemia	0 (0)		0 (0)		1.000	0 (0)		5 (0.8)		1.000
Lymphoma	0 (0)		1 (4.0)		1.000	1 (2.4)		10 (1.6)		0.505
Moderate-severe liver disease	0 (0)		1 (4.0)		1.000	1 (2.4)		11 (1.8)		0.535
Metastatic solid tumor	0 (0)		0 (0)		1.000	0 (0)		7 (1.1)		1.000
AIDS	0 (0)		0 (0)		1.000	0 (0)		0 (0)		1.000
Mean Charlson index	2.31	1.54	2.88	1.56	0.258	2.66	1.56	1.50	1.93	<0.001

AIDS: acquired immunodeficiency syndrome.

**Table 3:** Capsule endoscopy results.

Characteristics	*N* (%)
CE system	
PillCam	29 (70.7)
EndoCapsule	10 (24.4)
MiroCam	2 (4.9)
CE results	
Negative	16 (39.0)
Positive	25 (61.0)
Type of positive findings	
Mass lesion	3 (12.0)
Blood on CE	4 (16.0)
Erosions/ulcerations	9 (36.0)
Angiodysplastic/vascular lesions	9 (36.0)
Location of findings	
Esophagus	0 (0.0)
Stomach	3 (12.0)
Small bowel	21 (84.0)
Large bowel	1 (4.0)

CE: capsule endoscopy.
